# Molecular subtypes based on Wnt-signaling gene expression predict prognosis and tumor microenvironment in hepatocellular carcinoma

**DOI:** 10.3389/fimmu.2022.1010554

**Published:** 2022-10-06

**Authors:** Weifeng Xu, Caiyun Nie, Huifang Lv, BeiBei Chen, Jianzheng Wang, Saiqi Wang, Jing Zhao, Yunduan He, Xiaobing Chen

**Affiliations:** ^1^ Department of Medical Oncology, Affiliated Cancer Hospital of Zhengzhou University, Henan Cancer Hospital, Zhengzhou, China; ^2^ State Key Laboratory of Esophageal Cancer Prevention & Treatment, Zhengzhou University, Zhengzhou, China

**Keywords:** prognosis, tumor microenvironment, Wnt β-catenin signaling, hepatocellular carcinoma, TCGA (The Cancer Genome Atlas Program)

## Abstract

Based on increasing research evidence, hepatocellular carcinoma (HCC) is heterogeneous, and genetic profiling has led to the identification of multiple subtypes of this disease. To advance our knowledge and the ability to use individualized medicine in the treatment of HCC, it is essential to perform a complete and methodical characterization of various molecular subtypes. The canonical Wnt/β-catenin pathway is an evolutionarily conserved complicated signaling mechanism that plays a role in carcinogenesis and progression of HCC. In this study, we acquired RNA sequencing, somatic mutation, and clinical data from 701 patients from The Cancer Genome Atlas and Gene Expression Omnibus databases and stratified patients into two subgroups: WNT-high and WNT-low. In general, the WNT-high subtype is associated with an immunosuppressive microenvironment, poor prognosis, cancer-related pathways, and a low response to immune checkpoint therapy. We also found that WNT3 is negatively linked to CD8^+^ T-cell infiltration using multiple immunofluorescence assays. Finally, we developed a WNT-related prognostic model to predict the survival time of patients with HCC. In summary, we developed a new classification scheme for HCC based on Wnt signaling signatures. This classification produced substantial clinical effects, both in terms of assessing patient prognosis and immunotherapy administered to patients with HCC.

## Introduction

Liver cancer is the fourth leading cause of cancer-related mortality and the sixth most prevalent contributor to cancer morbidity worldwide (1). Hepatocellular carcinoma (HCC) is responsible for the majority of primary liver malignancies. Although its diagnosis has improved owing to advances in imaging techniques, the prognosis is still dismal, with a 5-year survival rate of <20%, and the choices available for treating HCC are limited (1, 2). The development of next-generation sequencing techniques and their widespread availability has given us the opportunity to investigate and record not only the specific genetic alterations of HCC cells but also the specific compositions of the various cell types found in the tumor microenvironment (TME) and their interplay with HCC cells at a certain level that was not previously possible. Therefore, the precise classification of patients with HCC into certain cancer types based on high-sensitivity genetic sequencing may help improve the clinical outcomes.

The canonical Wnt/β-catenin pathway is a complex signaling system that is evolutionarily conserved and affects basic physiological and pathological functions (3). This pathway is implicated in the maintenance of hepatic homeostasis and the development of distinctive hepatic properties, including metabolic zonation and regeneration in a mature healthy liver (3, 4). In HCC, Wnt/β-catenin signaling is often hyperactivated, which subsequently contributes to tumor growth and invasion (5−6). Interestingly, the Wnt/β-catenin pathway was recently characterized as playing a role in modulating the infiltration of immune cells in the TME, which has become a new research interest because of its possible influence on responsiveness to immunotherapy regimens (7−8). The practice of personalized medicine and the creation of innovative treatment strategies may benefit from targeting the Wnt/β-catenin signaling pathway.

In this study, we hypothesized that the molecular subtypes classified by Wnt/β-catenin signaling would exhibit distinct clinical and pathological features, prognostic factors, and TME. This study aimed to (i) identify the molecular subtypes of HCC based on Wnt/β-catenin signaling, (ii) analyze the prognostic value, anti-tumor immunity, and TME among these subtypes, and (iii) construct and validate a WNT-related prognostic model.

## Materials and methods

### Datasets

The Cancer Genome Atlas (TCGA) database was searched for RNA sequencing, somatic mutations, and relevant clinical data from 365 patients with HCC (https://portal.gdc.cancer.gov/). Similar data were also acquired from 336 patients with HCC in Gene Expression Omnibus (GEO) database to act as verification datasets. The accession number of GEO datasets was GSE14520 and GSE76427 (9, 10).

### Integration of protein−protein interaction network

We used the STRING database to create a PPI network, Cytoscape (https://cytoscape.org/), a platform that uses open-source software for the visualization of complicated networks, and the integration of these networks with any type of attribute data. We created a PPI network using Cytoscape and then used this network to examine the interaction relationships of the key genes involved in Wnt signaling-associated genes.

### Consensus clustering

Consensus clustering was undertaken to ascertain the molecular subtypes associated with Wnt signaling *via* the “ConcensusClusterPlus” package in R software. Subsequently, the ideal cluster numbers between k = 2 and k = 10 were evaluated, and to ensure that the outcomes would be consistent and easy to reproduce, this method was carried out one thousand times. A cluster map was generated using the pheatmap tool in R.

### Principal component analysis

PCA was conducted to evaluate the similarities and differences in transcription patterns across the various types. After loading the gene names together with the associated expression values and sample data, the “limma” package of the R program was employed to perform the analysis. The results were displayed using the “ggplot2” package.

### Immune cell type fractions estimation

CIBERSORT was conducted to ascertain the number of 22 different types of immune cells that were present in each HCC specimen. In the CIBERSORT system (https://cibersort.stanford.edu/), the differentiation of 22 different immune cells was accomplished with the use of a leukocyte gene matrix that contained 547 genes. These immune cells comprise resting NK cells, activated NK cells, gamma delta T cells, monocytes, follicular helper T cells, regulatory T cells (Tregs), resting CD4 memory T cells, activated CD4 memory T cells, CD8^+^ T cells, naïve CD4^+^ T cells, naïve B cells, memory B cells, plasma cells, macrophages (M0, M1, M2), eosinophils, neutrophils, activated mast cells, resting mast cells, resting dendritic cells, and activated dendritic cells. To further assess the reliable results of immune score evaluation, we used “immuneeconv” package to estimate immune cell scores based on TIMER and MCP-counter algorithms. The ssGSEA algorithm was completed using the “GSVA” and “GSEABase” packages in R.

### Establishment of the WNT prognostic signature

Following the univariate Cox regression analysis, a LASSO Cox regression analysis was conducted on the statistically significant WNT signaling-associated genes to determine the particular coefficient values for each association. The LASSO method of regression analysis is a technique for enhancing the accuracy of predictions and the interpretability of the generated statistical model by performing both variable selection and regularization. Therefore, LASSO Cox regression is an excellent choice for building a prognostic model based on gene expression patterns.

Comparison of the OS rates between the low- and high-risk groups was performed using Kaplan−Meier analysis, which was conducted in R using the survival and Survminer packages.

### Prediction of response to immunotherapy

To assess the immune checkpoint blockade (ICB) responsiveness, a tumor immune dysfunction and exclusion (TIDE) investigation was performed. Jiang et al. developed this analytical method (TIDE) that predicts ICB responsiveness by employing the two most important strategies that tumors use to evade the immune system: T-cell dysfunction induced in tumors that have high infiltrating levels of cytotoxic T lymphocytes (CTLs) and suppressed T-cell infiltration in tumors that have a low level of CTLs.

### Multiple immunofluorescence

Tissue microarrays of 36 HCC specimens were acquired from Shanghai Outdo Biotech (Shanghai, China) and used to conduct additional research on the link between WNT3 expression and CD8+ T cell presence in the HCC-TME. The multiplex immunohistochemistry (IHC) experiment was performed by employing staining cycles in the following order. Specifically, after deparaffinization, tissue slices of HCC that had been fixed in formalin and embedded in paraffin were subjected to microwave treatment in citrate for antigen retrieval. Next, the sections were blocked in normal goat serum at a concentration of 10% before incubating overnight with primary antibodies: rabbit anti-WNT3 antibody (1:200, ab32249, Abcam) and mouse anti-CD8 antibody (1:100, ab17147, Abcam). The sections were left for a thirty-minute incubation period at ambient temperature with the corresponding horseradish peroxidase-conjugated secondary antibodies (Abcam, CN). The tyramide signal amplification dye was used to display the antigenic binding sites. Each antibody was labeled with cy3-tyramide (1:1,000, G1235, Servicebio) and fluorescein isothiocyanate-tyramide (1:1,000, G1235, Servicebio). The positive percentage of CD8 T cells was calculated using Indica Labs-HighPlex FL module (v3.1.0) of Halo analysis software (Indica Labs, USA). The mean fluorescence intensity of WNT3 was quantified by Indica Labs-Highplex FL (v3.1.0) module of Halo software (Indica Labs, USA). The correlation between WNT3 expression and CD8^+^ T cell infiltration was calculated by Pearson correlation in R software

### Statistical analysis

The overall survival (OS) rates across various groups were compared *via* Kaplan−Meier analysis using Survminer and survival packages in R. The Kruskal−Wallis and Wilcoxon signed-rank tests were used to evaluate potential variations across the subtypes. Univariate Cox analysis was performed to determine the potential prognostic markers. Using the survivalROC R package, a receiver operating characteristic (ROC) curve was plotted to verify the accuracy of the risk model in the prediction of patients’ OS. The R software (version 3.5.2) was used for all statistical analyses.

## Results

### Identification of Wnt signaling-based subtypes by consensus clustering in HCC

**Figure 1:**
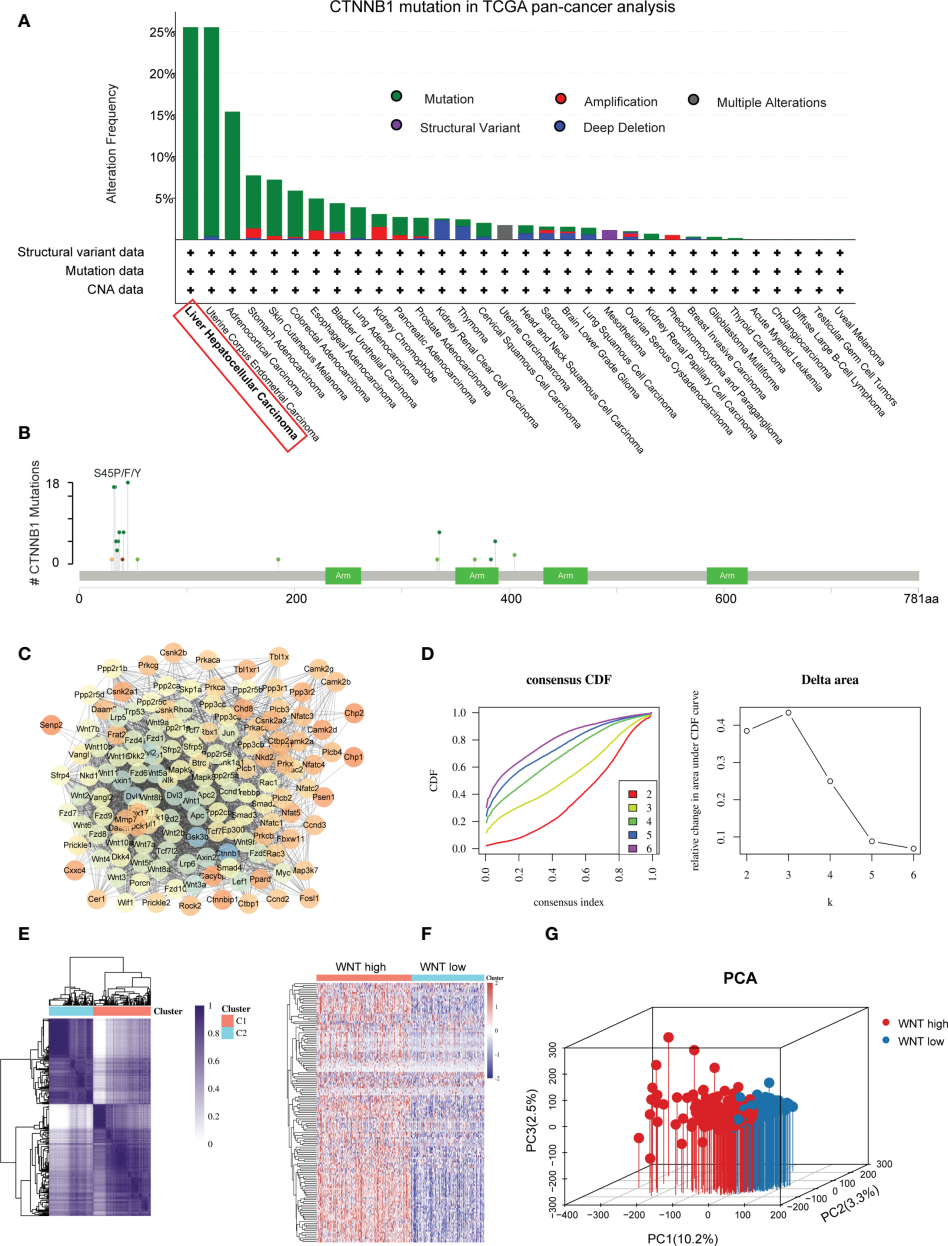
Identification of Wnt-based subtypes in HCC. **(A)** The bar plot presenting CTNNB1 mutation in TCGA pan-cancer dataset. **(B)** Amino acid mutation site of CTNNB1. **(C)** Protein−protein interactions of the Wnt signaling genes. **(D)** Delta area curve of consensus clustering. **(E)** Consensus clustering solution (k = 2) for Wnt signaling in HCC samples. **(F)** Heatmap of Wnt signaling gene expression in different clusters. **(G)** Principal component analysis plots.

We initially investigated alterations in Wnt signaling using TCGA pan-cancer datasets. Among these, CTNNB1 has the highest mutation rate in HCC. These results indicate that the Wnt/β-catenin pathway may play an essential role in carcinogenesis of HCC (**Figures 1A, B**). Next, we downloaded the Wnt-signaling gene set from the Gene Set Enrichment Analysis (GSEA) (KEGG_WNT_SIGNALING_PATHWAY.v7.5.1). We employed the STRING database to undertake a PPI network analysis to gain a deeper understanding of the mechanism by which these genes are involved in Wnt signaling (**Figure 1C**). We further determined Wnt signaling-based clusters in HCC using consensus clustering. After k-means clustering, we identified two clusters in TCGA cohort with distinct Wnt signaling-related gene expression patterns (**Figures 1D, E**). The expression levels of WNT genes varied among the different clusters. Overall, cluster C1 showed the highest Wnt signaling gene expression levels and was therefore defined as the WNT-high subtype. In contrast, cluster C2 displayed the lowest expression levels and was hence referred to as the WNT-low subtype (**Figure 1F**). PCA was performed to compare transcription patterns across various subtypes. In general, the results of PCA revealed that the samples from the two groups were well differentiated from one another, which suggested that both subtypes had unique transcriptional profiles (**Figure 1G**).

We further validated the repeatability of WNT-based classification in independent sample cohorts (GSE14520). Similarly, patients in the GEO cohort were stratified into WNT-low and WNT-high subtypes (**Supplementary Figure 1A**).

### Patients stratified into different WNT subtypes presented variant prognosis and clinicopathologic features

**Figure 2:**
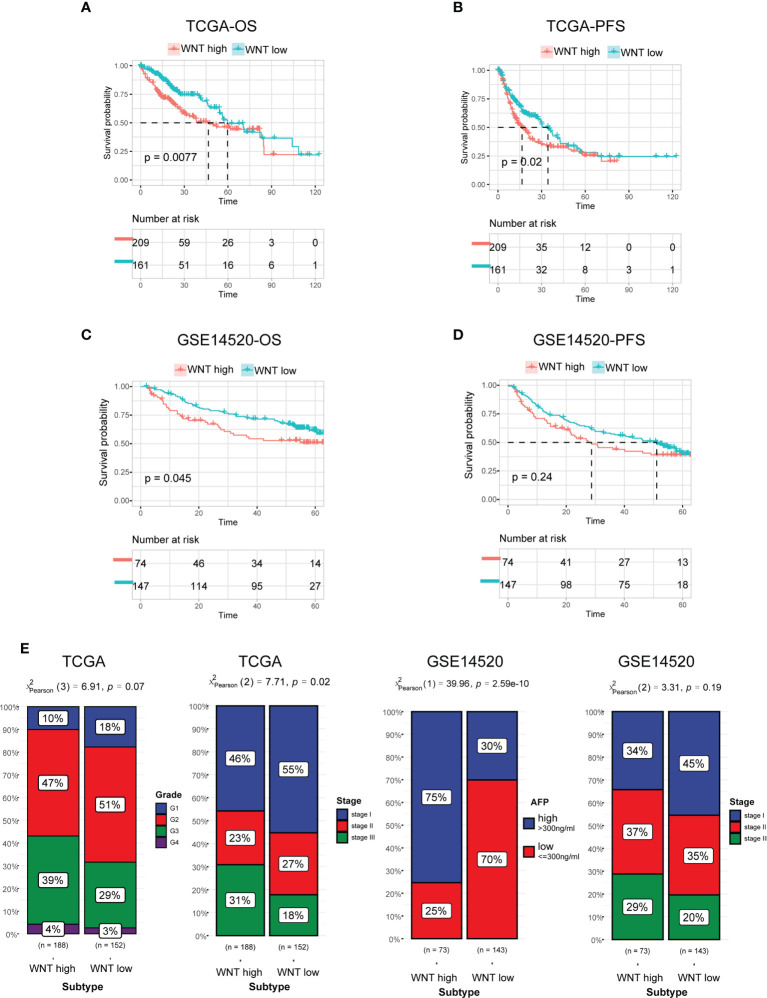
Prognosis and clinicopathologic characteristics between the Wnt subtypes. **(A, B)** Kaplan−Meier curves for patients with HCC classified into WNT-low and -high subtypes in TCGA in terms of OS **(A)** and PFS **(B)**. **(C, D)** Validation of Kaplan−Meier curves in the GEO dataset in terms of OS **(C)** and PFS **(D)**. **(E)** Bar plot presenting the clinicopathologic features of these subtypes.

Previous studies have shown that WNT signaling performs decisive functions in HCC tumor development. In accordance with these findings, survival studies have demonstrated that patients with different WNT-based subtypes have significantly different clinical outcomes. In general, the WNT-high subtype exhibited an unfavorable prognosis with the shortest OS and progression-free survival (PFS) (**Figures 2A, B**). In contrast to the WNT-high subtype, the WNT-low subtype was associated with the most satisfactory clinical outcomes. These findings were subsequently confirmed by analyses of the GEO cohort (**Figures 2C, D**). We further defined 3 or 4 subtypes by Consensus clustering but did not obtain the statistical significance in terms of the prognosis and failed to validate in external cohort GSE14520 (supplementary **Figures 2A, B**). Therefore, 2 subtypes could be ideal. We next compared the clinicopathological features of the subtypes. Patients stratified into WNT-high subtypes were associated with high grade, stage, and alpha-fetoprotein levels, which is in contrast to WNT-low subtypes (**Figure 2E**).

### WNT-based subtypes present a distinct TME

**Figure 3:**
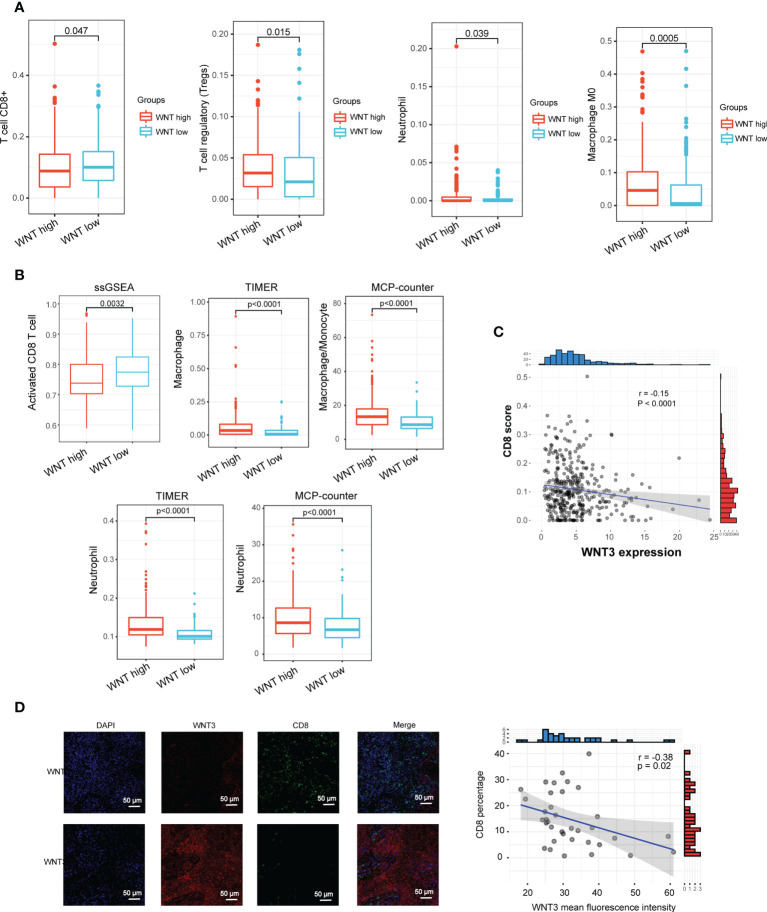
WNT-based subtypes are associated with the distinct tumor microenvironment. **(A)** Box plots presenting the infiltration score of CD8 T cells, Tregs, neutrophils, and macrophages. **(B)** Estimation of immune cell type fractions using different algorithms including ssGSEA, TIMER and MCP-counter. **(C)** Correlation between CD8 score and WNT3 expression. **(D)** Multiplex immunofluorescence validated the correlation between CD8^+^ T cell infiltration and WNT3 expression.

**Figure 4:**
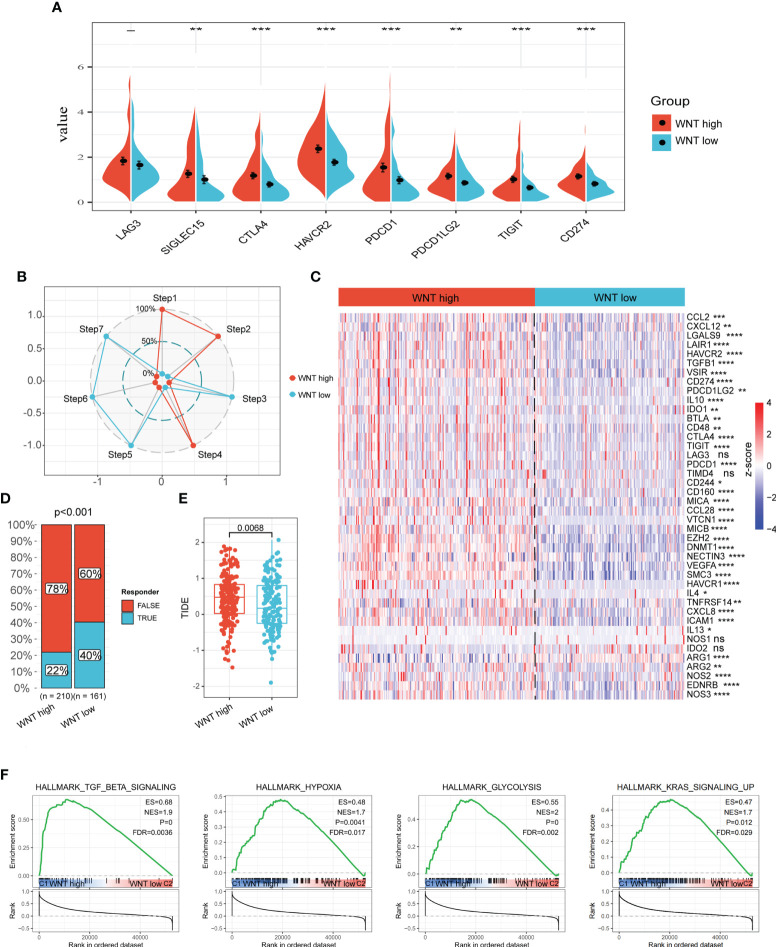
WNT-high subtypes are associated with the immune suppressive tumor microenvironment. **(A)** Violin plots of immune checkpoint expression. **(B)** Estimated score of the seven-step cancer-immunity cycle. **(C)** Heatmap of gene expression associated with the negative regulation of the immune processes. **(D)** Bar plot of ICB response rate. **(E)** Box plot of TIDE score. **(F)** GSEA plot of the underlying biological processes associated with WNT subtypes. (ns, p > 0.05; *, p < 0.05; **, p < 0.01; ***, p < 0.001; ****, p < 0.0001).

The newly revealed significance of the Wnt/β-catenin pathway in modulating immune cell infiltration into the TME has sparked fresh interest in this topic. Within the scope of this study, we investigated the TME characteristics across various tumor subtypes. In general, there was no remarkable difference between the WNT-high and WNT-low subtypes in terms of either immune score or tumor purity (**Supplementary Figure 3A**). The CIBERSORT method was used to determine immune heterogeneity among these subtypes. **Supplementary Figure 3B** summarizes the landscape of 22 (infiltrating) immune cells. In particular, patients with the WNT-high subtype exhibited substantially elevated proportions of immunosuppressive cells (Tregs, neutrophils, and macrophages), but significantly lower proportions of CD8^+^ T cells (**Figure 3A**). Similar to CIBERSORT results, ssGSEA validated a lower proportion of CD8^+^ T cells in WNT high subtype, and TIMER and MCP-counter verified higher proportions of immunosuppressive cells (neutrophils and macrophages) in WNT high subtype (**Figure 3B**). WNT3 is a critical molecule involved in WNT signal transduction. We further explored the correlation between WNT3 expression and CD8^+^ T cell infiltration. In TCGA database, WNT3 expression was negatively correlated with CD8 T cell score (**Figure 3C**). To further validate the association between WNT3 and CD8^+^ T cells in HCC, we performed multiplex immunofluorescence analysis. In line with the results from database analysis, multiplexed immunofluorescence analysis showed that high WNT3 expression was associated with low CD8^+^ T cell levels in the TME (**Figure 3D**). In addition, most immune checkpoints were elevated in the WNT-high subtype (**Figure 4A**). Conversely, the WNT-low subtype exhibited an opposite trend. These results illustrate that immunosuppressive cells may drive the immunosuppressive microenvironment of the WNT-high subtype.

The “cancer-immunity cycle” is a conceptualization of anti-tumor immunity that may be broken down into seven sequential processes, which include the following: release of tumor antigens (step 1), tumor antigen presentation (step 2), priming and activation (step 3), trafficking of T cells to tumors (step 4), infiltration of immune cells into tumors (step 5), recognition of tumor cells by T cells (step 6), and killing of tumor cells (step 7). We used TIP (a web-based platform that can resolve tumor immunophenotype profiling issues) to evaluate the anticancer immune activity of the seven-step cancer-immunity cycle among the three subtypes. Although the WNT-high subtype presented the highest activity in steps 1, 2, and 4, great attenuation of steps 5, 6, and 7 was observed (**Figure 4B**). These results indicate that mitigation of the immunosuppressive microenvironment in the WNT-high subtype may contribute to good clinical outcomes in HCC. In addition, the WNT-high subtype had the greatest number of upregulated genes implicated in the immunosuppressive modulation of immune processes, followed by the WNT-low subtype (**Figure 4C**).

We subsequently employed TIDE (a computational approach designed to derive the possibility of immune evasion by tumors based on the gene expression patterns of tumor tissues) to investigate the possibility of immunotherapy being effective in clinical settings for certain subtypes. As per the findings of this study, the WNT-high subtype exhibited a decreased response rate in contrast with the WNT-low subgroup, which suggests that patients with the WNT-high subtype are not candidates for immunotherapy (**Figures 4D, E**). Moreover, we analyzed the underlying pathways that correlated with the subtypes. GSEA revealed that the WNT-high subtype experienced substantial enrichment in the negative modulation of the immune pathway, including TGF-β signaling, hypoxia, glycolysis, and KRAS signaling (**Figure 4F**).

According to these findings, patients with the WNT-high subtype have a great possibility of developing an immunosuppressive microenvironment as a direct consequence of the up-modulation of immunosuppressive cytokines, the expression of immune checkpoints, and the infiltration of immunosuppressive cell populations, which may ultimately contribute to poor prognosis.

### Establishment and verification of the WNT-related prognostic signature

**Figure 5:**
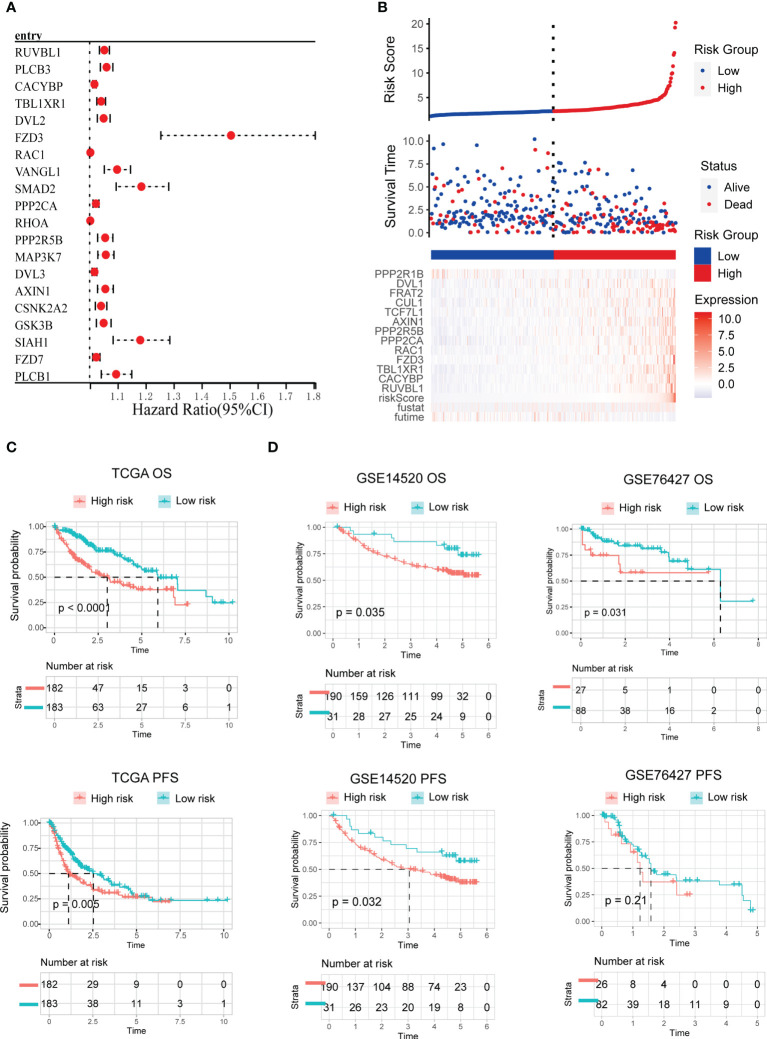
Development and validation of the WNT-related prognostic signature. **(A)** Univariate cox analysis of WNT-related genes associated with overall survival. The top ten genes with the most significant p-value are presented. **(B)** Risk scores distribution, survival status of each patient, and heatmaps of prognostic 13-gene risk signature. **(C, D)** Kaplan−Meier curves for patients with high- or low-risk scores in TCGA training cohort **(C)**, GEO testing cohort **(D)**.

We created a prognostic model based on WNT signaling genes. In univariate Cox analysis, 66 of the 150 WNT genes were strongly linked to OS. **Figure 5A** summarizes the top ten genes with the most significant p-values. Subsequently, 66 WNT genes identified by Cox univariate analysis were evaluated and chosen for the prediction model in the LASSO regression analysis. The following equation was used to develop the risk score model: risk score = (0.0116)*RUVBL1 + (0.00454)*CACYBP + (0.01230)*TBL1XR1 + (0.1157)*FZD3 + (0.0004)*RAC1 + (0.0001)*PPP2CA + (0.0113)*PPP2R5B + (0.0015)*AXIN1 + (0.0068)*TCF7L1 + (0.00059)*CUL1 + (0.00371)*FRAT2 + (0.00065)*DVL1 + (-0.0033)*PPP2R1B. Genes included in the final model also showed statistical significance in multivariate Cox analysis (**Supplementary Figure 4**). Furthermore, we evaluated the association between the risk score and survival status. As per the findings of our study, the low-risk cohort had a substantially greater number of alive statuses than the high-risk cohort (**Figure 5B**). The prognostic value of this risk model was additionally evaluated using Kaplan–Meier analysis. Overall, the high-risk score was linked to unfavorable OS and PFS in TCGA training cohort (**Figure 5C**), which was subsequently verified in the GSE14520 and GSE76427 testing cohorts (**Figure 5D**).

### WNT risk signature demonstrates the high predictive potential for prognostic evaluation

**Figure 6:**
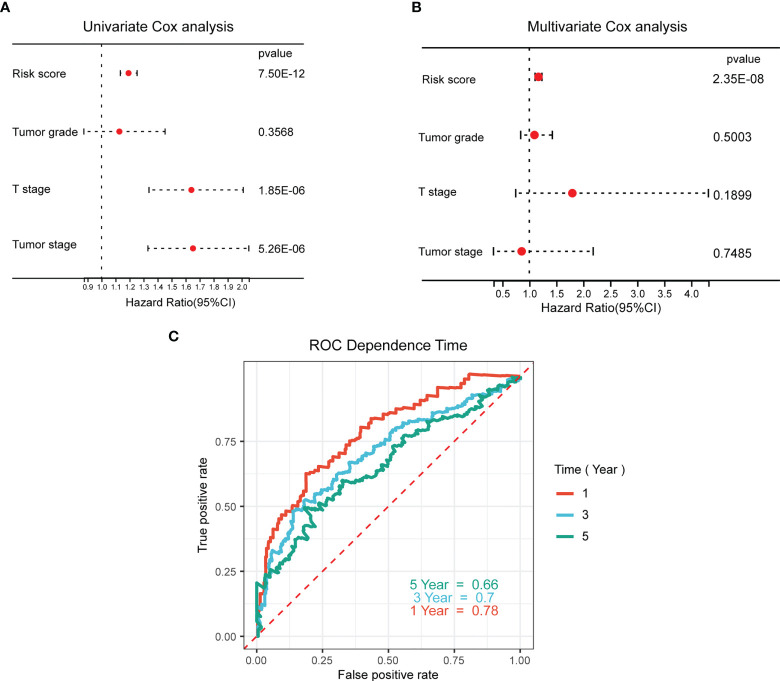
Prognostic value of the WNT-associated risk signatures in HCC samples. **(A, B)** Univariate **(A)** and multivariate **(B)** Cox analyses of the independent prognostic value of the WNT-related signature in patients with HCC. **(C)** ROC curves of the predictive efficiency of the WNT risk signature on the 1-, 3-, and 5-year survival rate.

Univariate and multivariate Cox analyses were performed to determine the independent prognostic value of the Wnt signature with regard to OS. As illustrated by the findings of univariate analysis, a high WNT risk score was strongly associated with unfavorable OS (**Figure 6A**). Other factors associated with unfavorable survival were the T stage and tumor stage. According to the findings of the multivariate study, a high WNT risk score was independently associated with a considerably more unfavorable OS (**Figure 6B**). This suggests that it may be an independent factor in determining the prognosis of patients with HCC. Subsequently, we performed a ROC curve analysis to examine the degree to which the WNT risk signature was able to accurately predict the survival rates (predictive efficiency) over 1, 3, and 5 years. The area under the ROC curve (AUC) showed strong predictive power, with values of 0.78, 0.7, and 0.66 over 1, 3, and 5 years, respectively (**Figure 6C**).

## Discussion

In this study, our primary objective was to identify different subtypes of HCC based on Wnt signaling. Our results demonstrate that HCC might be classified into WNT-high and -low subtypes with distinct clinicopathological features, prognosis, and TME. We demonstrated that this classification was both predictable and also capable of being reproducible. Collectively, the WNT-high subtype presents a grim prognosis, with an immunosuppressive microenvironment and a high frequency of oncogene mutations. In contrast, the WNT-low subtypes were associated with the most favorable clinical outcomes with the immunoreactive microenvironment among these subtypes. Moreover, we developed and validated a WNT-related prognostic model that presents strong power for prognosis assessment.

Clinical progress has been made in the prediction of patient prognoses and the selection of cancer treatment using molecular classifications in conjunction with gene expression patterns, and the exact classification of oncogenesis has been made possible by recent advances in DNA sequencing and methylation array technology (11−12). In HCC, the discovery of numerous significant molecular markers, the most remarkable of which are *TP53* mutations, has enabled the development of a precise technique for classifying HCC with significant prognostic value (13−14). In addition to the *TP53* mutation, more recent research has uncovered a second significant mutation in these tumors called CTNNB1, which also affects the clinical prognosis of patients. In HCC, the Wnt/β-catenin pathway is often upregulated and linked to the maintenance of tumor-initiating cells, as well as medication resistance, tumor growth, and metastasis (6). In this study, we established WNT-based subtypes that categorized patients with HCC into WNT-low and WNT-high subtypes with distinct clinicopathological features, prognosis, and TME.

Wnt/β-catenin signaling, a highly evolutionarily conserved pathway, functions in multiple cellular processes, including proliferation, differentiation, migration, genetic stability, apoptosis, and stem cell renewal. The recently reported functions of the Wnt/β-catenin pathway in modulating immune cell infiltrates in the TME and immunotherapy have piqued attention (15). Tumor-intrinsic β-catenin signaling suppresses the mobilization of CD103^+^ DCs in melanoma, preventing antitumor immune function. Mechanistically, active β-catenin signaling causes the transcriptional inhibitor ATF3 to be expressed, which inhibits CCL4 expression (8). Immune evasion, as well as tolerance of anti-PD-1 treatment, is promoted by β-catenin stimulation in hepatocellular carcinoma (8). In addition, high TMB NSCLC tumors activated WNT/β-catenin signaling, which modulated chemokine ligand expression and subsequent immune cell infiltration. Blocking Wnt/β-catenin signaling rescued the effects of anti–PD-1 in high TMB tumors, leading to tumor clearance. These pieces of evidence highlight the significant influence of this pathway on immunotherapeutic treatment outcomes (16). In line with the evidence, the results of our research indicate that the WNT-high subtype is linked to a lower level of T cell gene expression and lower immunotherapy response. Our evaluations included descriptions of changes in the molecular pathways and gene expression associated with the immune response in these subtypes. Nevertheless, it should be noted that our findings require further validation *in vitro* or *in vivo*. Our findings should be interpreted with this limitation in mind.

The Wnt/β-catenin pathway also play an important role in tumor microenvironment remodel. Interactions between cancer cells and the tumor-associated macrophages (TAMs) have been demonstrated to be mediated by the Wnt/β-catenin signaling pathway. A previous study showed that interleukin-1β, released by TAMs, might enhance the presence of β-catenin through GSK3β phosphorylation in colon cancer cells, thereby preventing the β-catenin destruction complex from performing its normal functions (17). Snail, a soluble component of Wnt target genes, is responsible for stimulating IL-β secretion in macrophages by colorectal cancer cells (18). Moreover, Wnt/β-catenin signaling promotes Treg survival. Similarly, in our study, we identified Treg and macrophage scores enriched in the WNT-high subtype.

In summary, our research sheds light on the links between WNT-based subtypes and prognosis, as well as alterations in the immune TME in patients with HCC. These findings could be useful for developing immune therapy-based treatments for HCC patients in the future. We also developed and verified a WNT-associated prognostic signature that exhibited remarkable value in the prediction of OS in patients with HCC.

## Data availability statement

The original contributions presented in the study are included in the article/**Supplementary Material**. Further inquiries can be directed to the corresponding author.

## Ethics statement

The studies involving human participants were reviewed and approved by institutional review board of Shanghai Outdo Biotechnology. Written informed consent for participation was not required for this study in accordance with the national legislation and the institutional requirements.

## Author contributions

Conceptualization and methodology: WX, CN; writing: WX; statistic calculation and validation: WX, YH, and HL; review and approval of concept/methodology: BC and JW; editing: SW, and JZ; project administration and funding acquisition: XC. All authors contributed to the article and approved the submitted version.

## Funding

This work was supported by the Medical Science and Technique Foundation of Henan Province (No. LHGJ20200185 for W.-F. X), and Science and Technique Foundation of Henan Province (No. 212102310771 for W.-F. X).

## Acknowledgments

Our results were generated using some of the basic data from TCGA Research Network: https://www.cancer.gov/tcga.

## Conflict of interest

The authors declare that the research was conducted in the absence of any commercial or financial relationships that could be construed as a potential conflict of interest.

## Publisher’s note

All claims expressed in this article are solely those of the authors and do not necessarily represent those of their affiliated organizations, or those of the publisher, the editors and the reviewers. Any product that may be evaluated in this article, or claim that may be made by its manufacturer, is not guaranteed or endorsed by the publisher.

## Supplementary material

Supplementary Figure 1
**(A)** Validation of WNT-based subtypes in GEO dataset.

Supplementary Figure 2
**(A, B)** Kaplan−Meier curves for patients with HCC classified into three **(A)** or four **(B)** subtypes in TCGA in terms of OS and PFS.

Supplementary Figure 3
**(A)** Violin plots of immune score and tumor purity score. **(B)** The relative proportion of immune infiltration in HCC samples.

Supplementary Figure 4
**(A)** multivariate Cox analysis of genes included in the final model.
