# Supplementary figures and images for: Molecular subtypes based on Wnt-signaling gene expression predict prognosis and tumor microenvironment in hepatocellular carcinoma
Source: Front Immunol. 2022 Oct 6;13:1010554. doi: 10.3389/fimmu.2022.1010554 (PMC9582750; doi:10.3389/fimmu.2022.1010554)

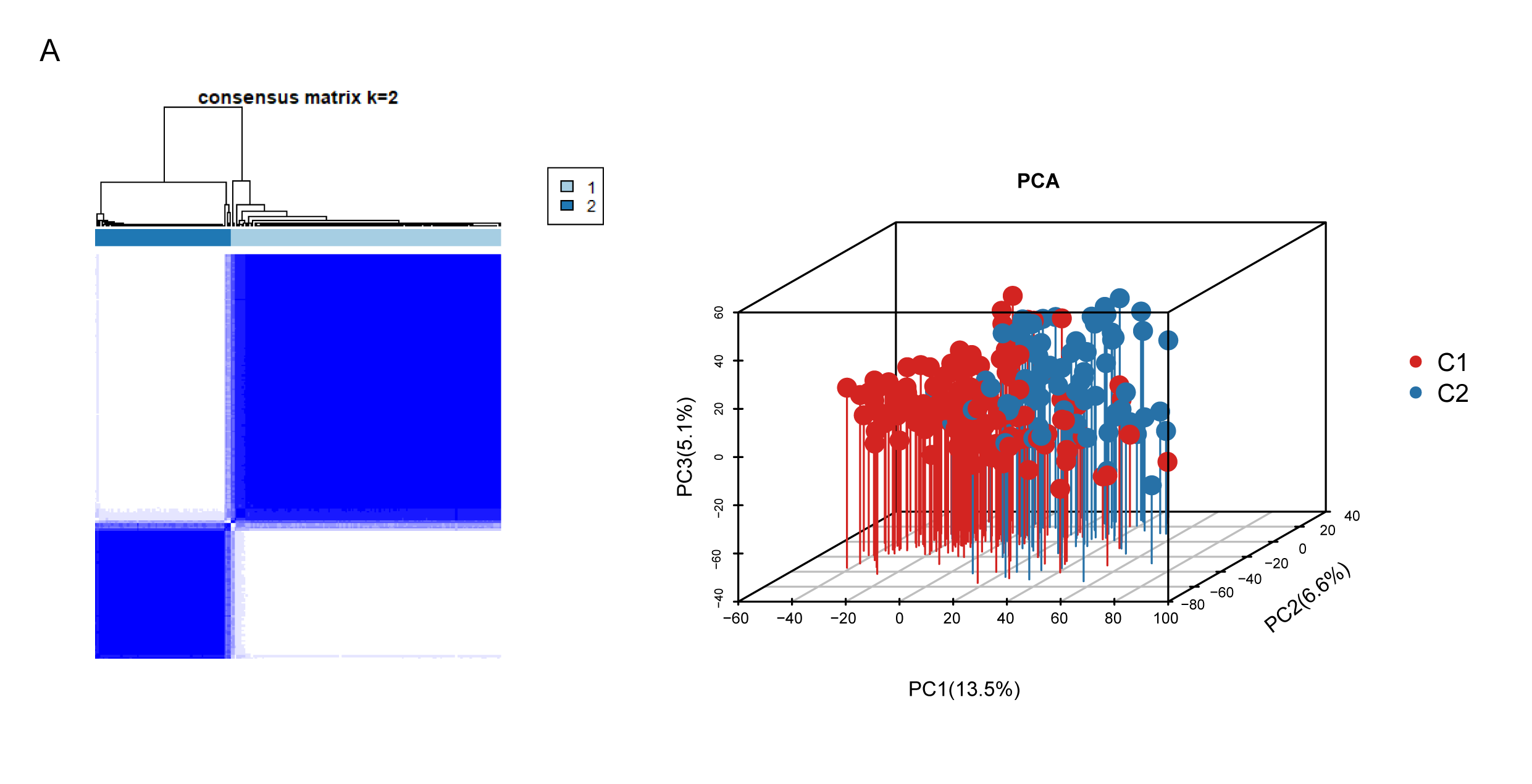

Supplement: Supplementary Figure 1 — (A) Validation of WNT-based subtypes in GEO dataset. [file Image_1.tif]

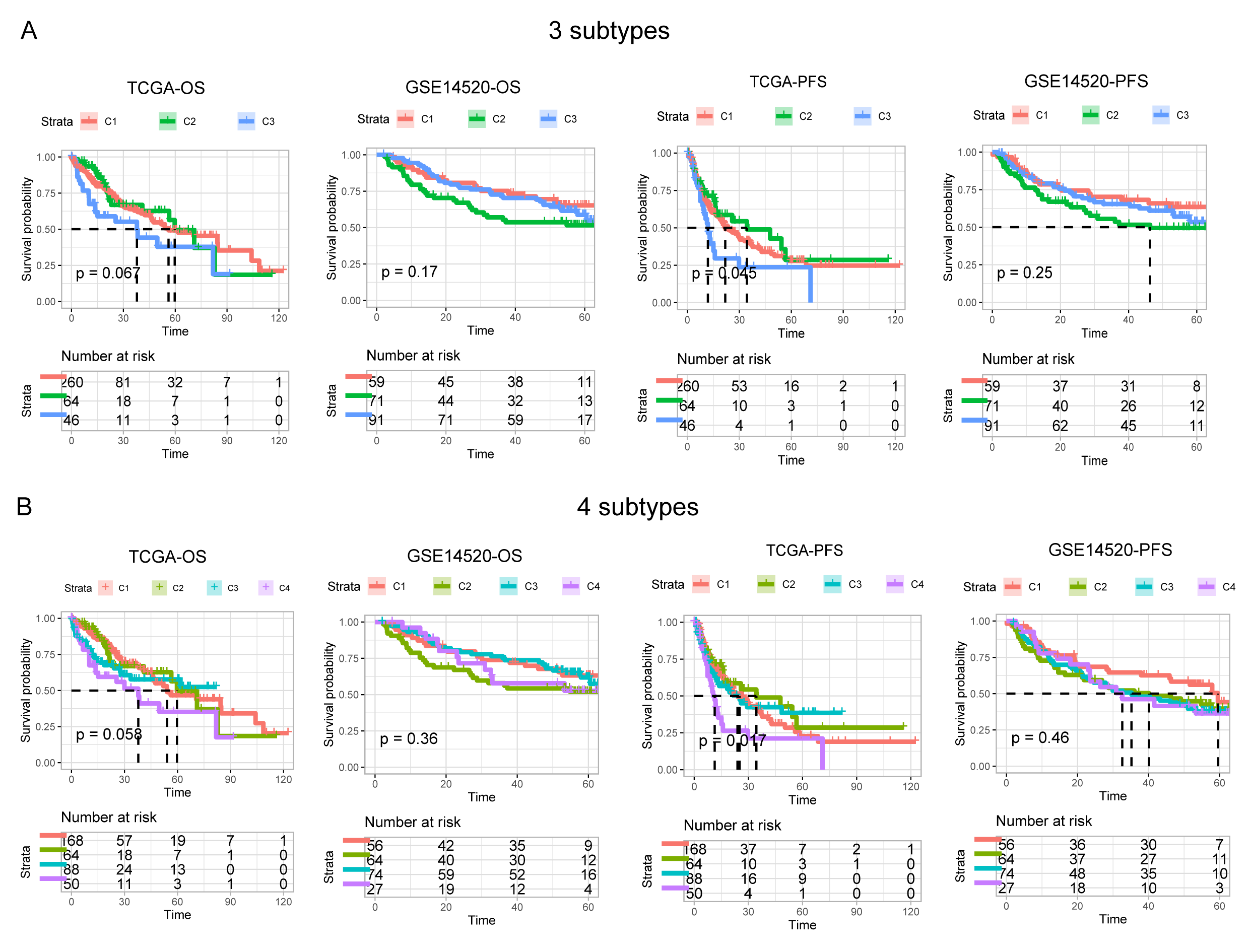

Supplement: Supplementary Figure 2 — (A, B) Kaplan−Meier curves for patients with HCC classified into three (A) or four (B) subtypes in TCGA in terms of OS and PFS. [file Image_2.tif]

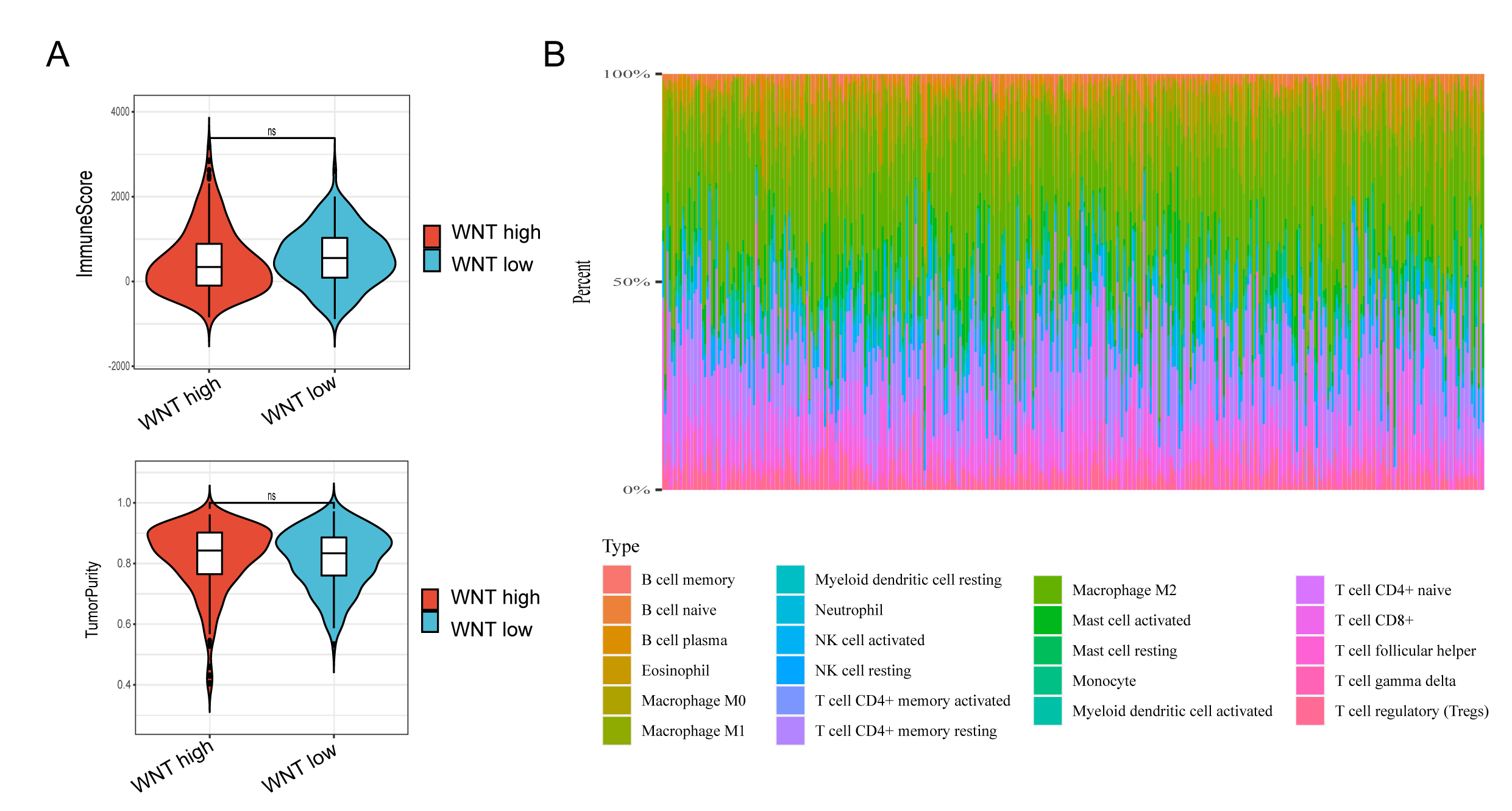

Supplement: Supplementary Figure 3 — (A) Violin plots of immune score and tumor purity score. (B) The relative proportion of immune infiltration in HCC samples. [file Image_3.tif]

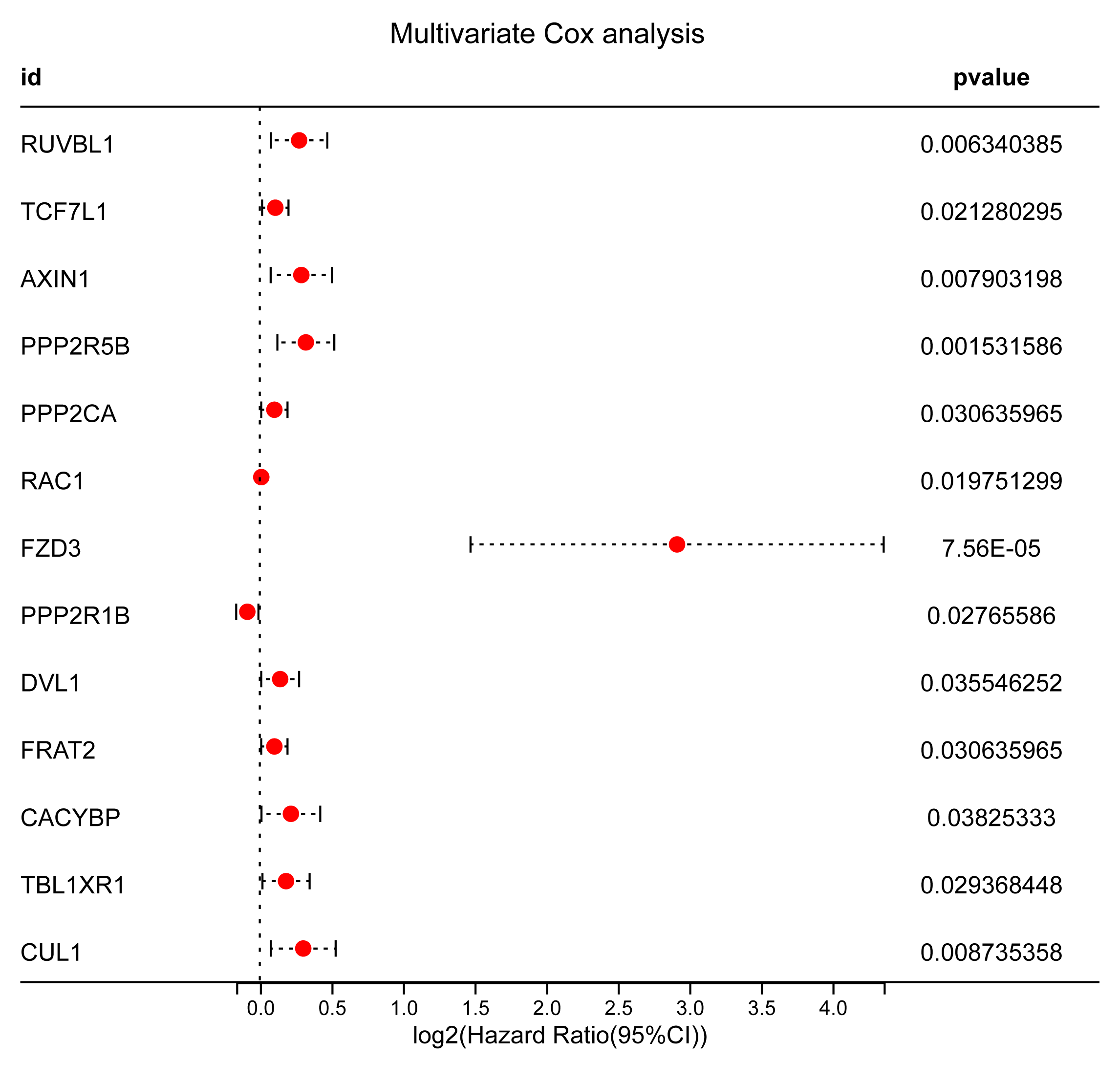

Supplement: Supplementary Figure 4 — (A) multivariate Cox analysis of genes included in the final model. [file Image_4.tif]
